# Subwavelength Metamaterial Unit Cell for Low-Frequency Electromagnetic Absorber Applications

**DOI:** 10.1038/s41598-018-35267-w

**Published:** 2018-11-13

**Authors:** Heijun Jeong, Toan Trung Nguyen, Sungjoon Lim

**Affiliations:** School of Electrical and Electronic Engineering, Chung-Ang University, Heukseok-Dong, Dongjak-Gu Seoul 06974 Republic of Korea

## Abstract

In this paper, we propose a subwavelength metamaterial unit cell for low-frequency electromagnetic absorber applications. To realize a periodic array for a metamaterial absorber, the footprint size and thickness of a unit cell must be miniaturized to a subwavelength. We achieved the electrical size of the unit cell as 0.027λ × 0.027λ × 0.043λ at 2.4 GHz by introducing the inductive lump elements to a symmetric square-loop resonator. The performance of the proposed absorber was demonstrated by full-wave simulations and measurements. An inductance tolerance of 2% yielded errors of 1.2% and 1.25% in the absorptivity and absorption frequency, respectively. A prototype with 13 × 27 unit cells was fabricated and its absorptivity was measured to be 99.6% at 2.4 GHz.

## Introduction

A metamaterial is a periodic structure in which an artificial structure is infinitely arranged^1^. These metamaterials are used in various electromagnetic (EM) fields because they are capable of controlling permittivity and permeability under numerous conditions, including EM cloaking^2–4^, electromagnetic interference (EMI)/electromagnetic compatibility (EMC) solutions^5,6^, human body applications^7^, super lenses^8–10^, and sound wave technologies^11–13^. Another metamaterial application is an EM absorber based on metamaterials, which was first reported by Landy^14^. Absorbers, prior to the metamaterial absorber, were primarily based on the classes of materials that comprised the absorber. The wedge-tapered absorber^15–19^, which is based on ferrite materials, possesses excellent absorption ability; however, its drawbacks include large thickness and high cost. The Jaumann absorber^20,21^ overcomes these disadvantages by employing a quarter wavelength (λ/4) thickness of a dielectric material and resistive sheet, which allows for a product that is thinner than a wedge-tapered absorber; however, this absorber is still regarded as bulky. In particular, a lower absorption frequency corresponds to a thicker absorber owing to the use of quarter wavelength thickness. Meanwhile, the metamaterial-based absorber^22–24^ can achieve excellent absorption with a small thickness because the EM wave is absorbed by the resonance structure. Moreover, the metamaterial absorber possesses the advantage of cost-effectiveness with an easy fabrication process.

Despite the advantages of the metamaterial absorber, because the wavelength increases at a low frequency, the metamaterial (MM) unit-cell size increases. In particular, the periodic implementation of the large unit cell becomes challenging in low-frequency and acoustic applications. Several methods have recently been proposed to achieve low-frequency MM absorbers, including the use of a capacitor-loaded structure^25^, multiple layers^26,27^, snake-shape structure^28^, magnetic rubber plate and cross resonator^29^, corrugated surface^30^, and a sandwiched metal-dielectric-metal structure^31^. These works indicate that it is difficult to obtain a simultaneously thin and small unit cell in a single layer.

Herein, we propose a small and thin electric MM absorber in a single layer by introducing an inductive lumped element to a square-loop resonator. Owing to the additional inductance, the absorption frequency is decreased without a corresponding increase in size. Four lumped elements are loaded on the square loop to retain the symmetrical geometry, which enables polarisation-independent performance. The proposed absorber is analyzed using a simplified equivalent transmission line model and full-wave simulations^24,32^. The absorptivity of the proposed absorber is experimentally verified by fabricating 13 × 27 unit cells. The proposed concept is described in the following sections.

## Results

### Design and Simulation

**Figure 1:**
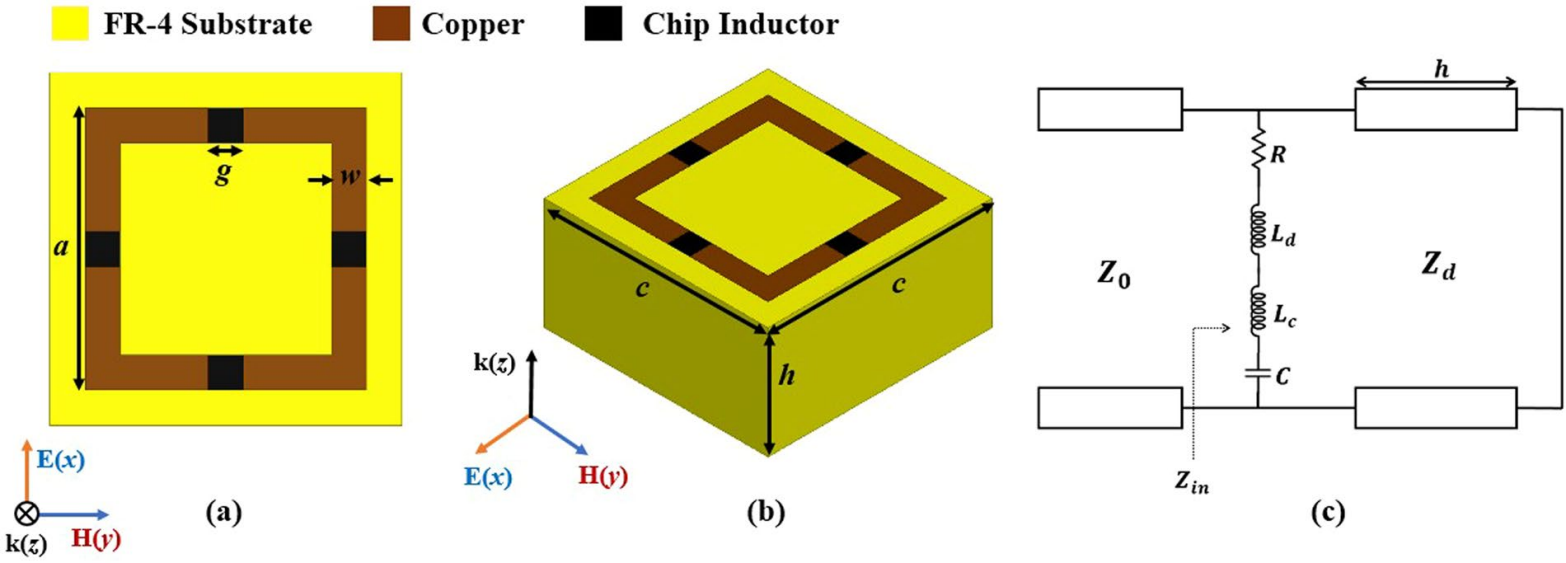
(**a**) Top view of the proposed unit cell structure. (**b**) Perspective view of the proposed unit cell structure. (**c**) Equivalent transmission line mode of the proposed unit cell.

The top and perspective views of the proposed MM unit cell are illustrated in Fig. 1(a,b), respectively. The MM unit cell is designed based on the square-loop resonator. The resonant frequency of the square-loop resonator is dependent on the effective inductance and capacitance. Therefore, the resonant frequency can be reduced by increasing the effective inductance. Herein, the lumped inductor is loaded onto the square-loop resonator. The four inductors are loaded on each side of the square loop to retain the symmetrical geometry; this causes the absorptivity to remain unchanged although the polarisation of the incident wave is changed.

The equivalent transmission line mode of the proposed unit cell is shown in Fig. 1(c). **Z**_0_ and **Z**_d_ denote the characteristic impedances of air and the dielectric substrate, respectively. The square-loop resonator can be represented as *R*, *L*, and *C* in series. *R* represents the Ohmic resistance at the transmission line. Herein, the inductance of the lumped element (*L*_c_) is added to the inductance of the distributed element (*L*_d_). Therefore, the total effective inductance is *L*_c_ + *L*_d_, and the resonant frequency (*f*_r_) is provided by^22^

1 $${f}_{r}=\frac{1}{2\pi \sqrt{({L}_{c}+{L}_{d})\times C}}=\frac{1}{2\pi \sqrt{{L}_{eff}\times C}}\mathrm{.}$$

The absorptivity of the absorber can be expressed as^33^

2 $$A(\omega )=1-{\rm{\Gamma }}(\omega )-T(\omega )=1-{|{S}_{11}(\omega )|}^{2}-{|{S}_{21}(\omega )|}^{2},$$

where *Γ*(*ω*) and *T*(*ω*) are the reflection and transmission coefficients, respectively, and can be calculated from the *S*-parameters, such as *S*_11_ and *S*_21_. Because the bottom layer is fully covered by the conductive plane, the transmission is zero. Therefore, the absorptivity can be simplified and perfect absorption can be achieved by minimising the reflection^34^. By controlling the effective permittivity *ε*_r_(*ω*) and permeability *μ*_r_(*ω*) of the MM, the effective impedance of the proposed MM absorber at the absorption frequency is provided by

3 $$Z(\omega )=\sqrt{\frac{{\mu }_{0}{\mu }_{r}(\omega )}{{\varepsilon }_{0}{\varepsilon }_{r}(\omega )},}$$

where *ε*_0_ and *μ*_0_ are the permittivity and permeability of free space, respectively. Under normal incidence, when *Z*(*ω*) is the same as ${Z}_{0}=\sqrt{{\mu }_{0}/{\varepsilon }_{0}}=377\,{\rm{\Omega }}$, a reflection coefficient of zero can be achieved by tailoring *ε*_r_ and *μ*_r_ to be identical.

For a full-wave EM analysis, we used the ANSYS high-frequency structure simulator (HFSS). The FR-4 substrate (thickness *h* = 5.6 mm), with a dielectric constant of 3.9 and a loss tangent of 0.02, was selected. The top and the bottom patterns are realized using copper with an electric conductivity of *σ* = 5.8 × 10^7^ S/m. The final geometrical parameters of the proposed unit cell are *a* = 3.5 mm, *w* = 0.5 mm, *c* = 4 mm, *g* = 0.5 mm, and *h* = 5.6 mm. For the lumped inductive elements, four inductors with *L*_c_ = 17 nH are loaded on the gaps of the square loop.

**Figure 2:**
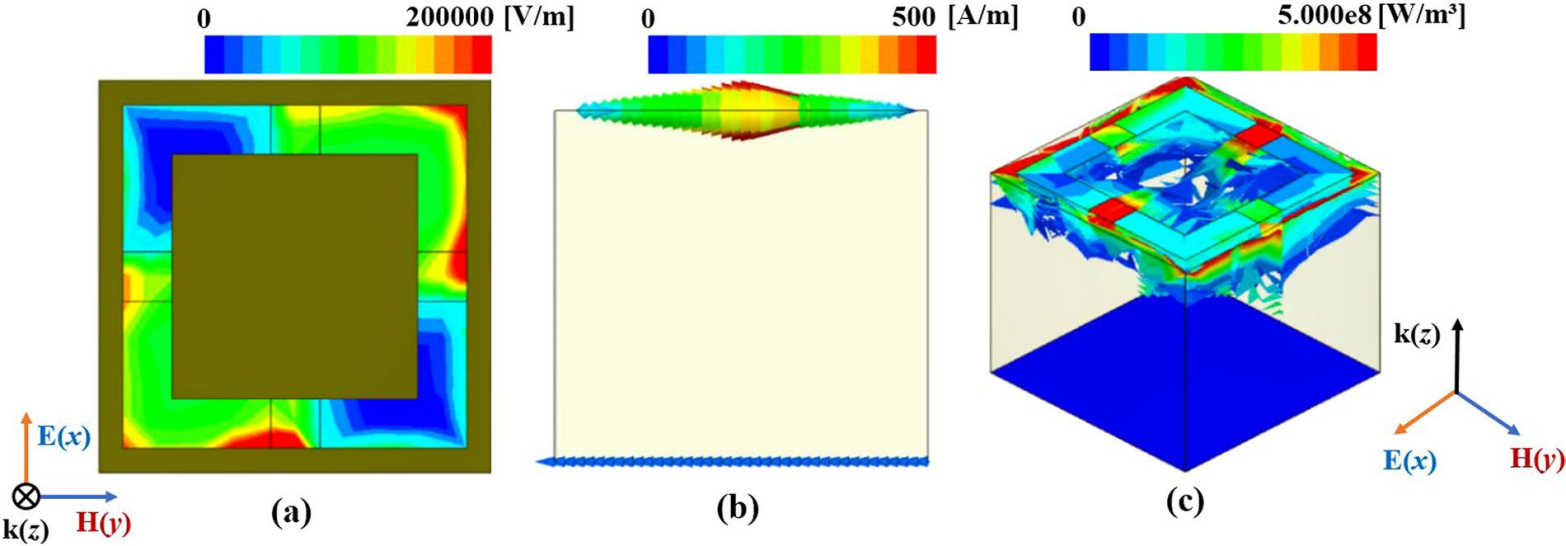
Simulated electric-field magnitude at 2.4 GHz. (**b**) Simulated surface-current vector at 2.4 GHz. (**c**) Simulated volume-loss magnitude at 2.4 GHz.

To demonstrate the electrical and magnetic responses of the proposed absorber, the electric-field magnitude and the electrical current–density vector are simulated. As shown in Fig. 2(a), the electrical resonance is generated from the outer rings and lumped inductors at 2.4 GHz. Owing to the strong resonance on the lumped inductors, the frequency shift effect is enhanced. In addition, the magnetic resonance is generated from the electric currents at the top and bottom layers, as shown in Fig. 2(b,c), respectively. These figures show that the EM energy transmitted to the substrate is dissipated as thermal losses owing to the dielectric loss of the FR-4 substrate and the resistive losses of the lumped inductors.

**Figure 3:**
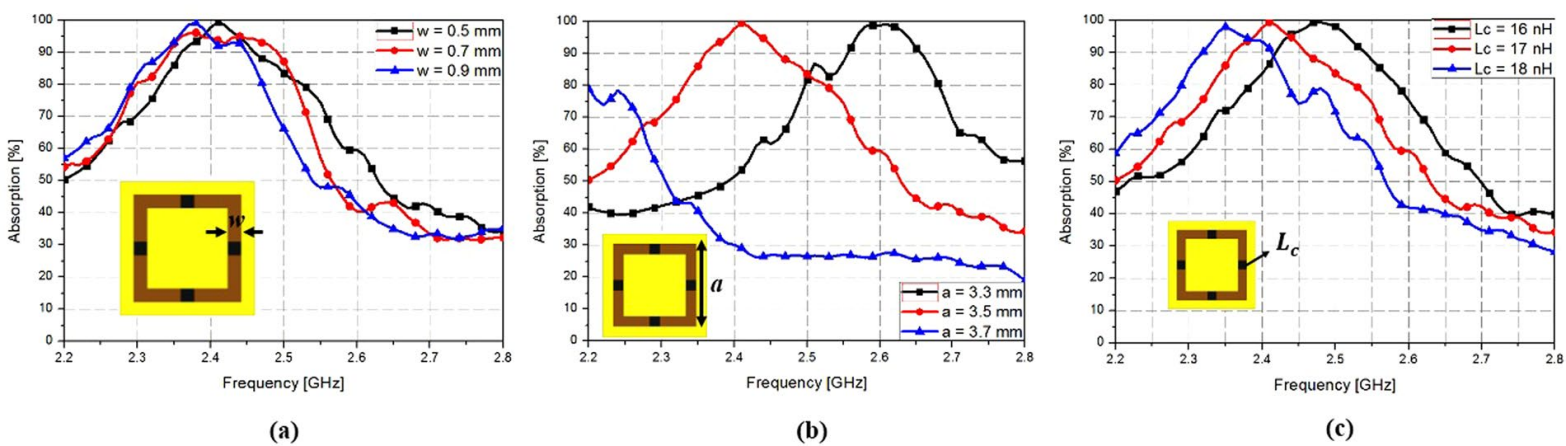
Simulated absorption bandwidth variation of the proposed MM absorber at different values of the parameters: (**a**) *w* varying from 0.5 mm to 0.7 mm, (**b**) *a* varying from 3.3 mm to 3.7 mm, (**c**) *L*_c_ varying from 16 nH to 18 nH.

Figure 3 shows the simulated absorption bandwidth variation of the proposed metamaterial absorber. Figure 3(a) shows the conductive square loop width (*w*) increase from 0.5 mm to 0.7 mm. When the width is 0.9 mm, the peak frequency is 2.35 GHz with 99% of the absorptivity. At a width of 0.7 mm, the peak absorption frequency slightly decreases and the absorptivity also decreases. However, when the width is 0.5 mm, the peak frequency is 2.4 GHz with 99% of the absorptivity. Thus, we choose a width of 0.5 mm to correspond with the peak frequency of 2.4 GHz with 99% of the absorptivity. Figure 3(b) shows that when the conductive square loop size (*a*) increases from 3.3 mm to 3.7 mm, the peak absorption frequency decreases from 2.65 GHz to 2.2 GHz. Further, Fig. 3(c) shows that when the chip inductor value (*L*_c_) is increased from 16 nH to 18 nH, the peak absorption frequency decreases from 2.6 GHz to 2.35 GHz. Herein, to set the absorption frequency at 2.4 GHz, we selected a conductive square loop size (*a*) of 3.5 mm and chip inductor value (*L*_c_) of 17 nH.

**Figure 4:**
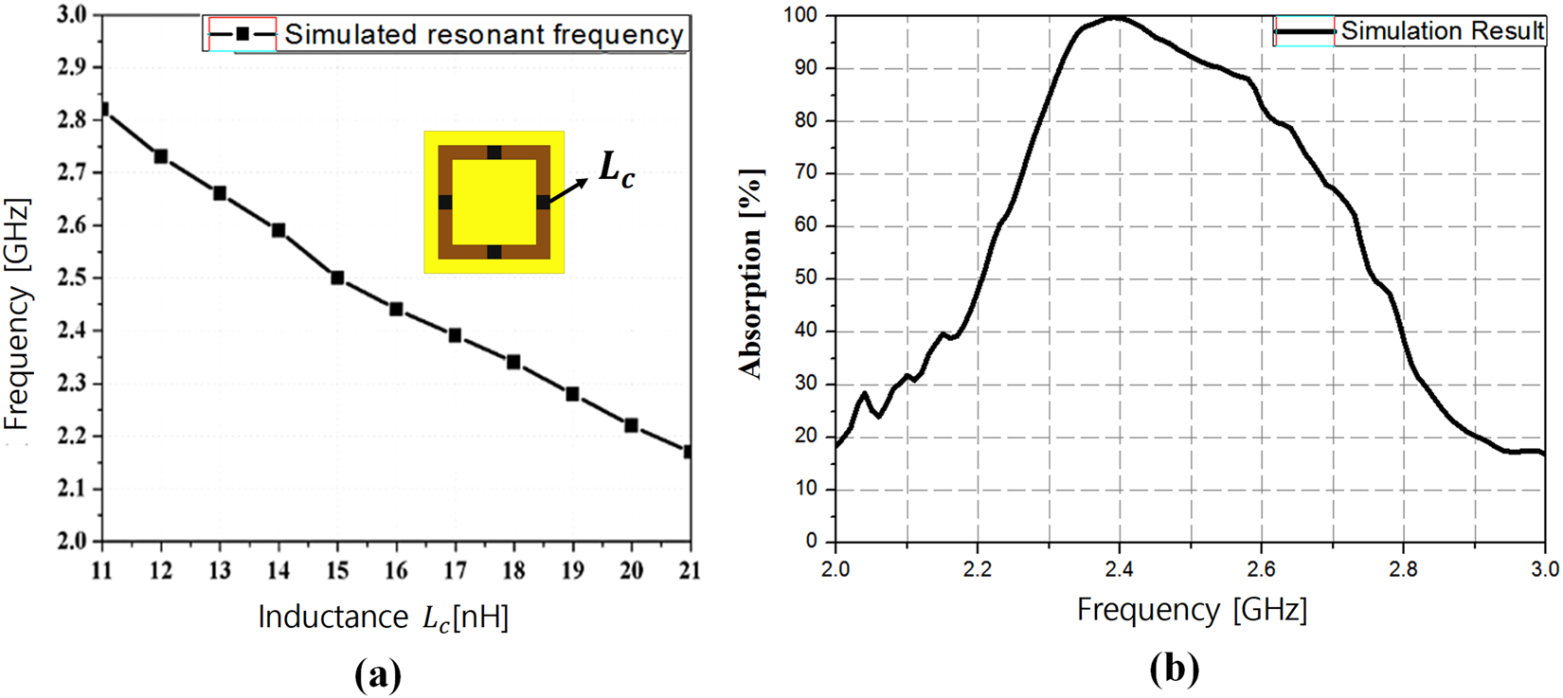
(**a**) Relationship between the resonant frequency and *L*_c_. (**b**) Simulated absorptivity of the proposed MM absorber.

The relationship between the resonant frequency and inductance of *L*_c_ is plotted in Fig. 4(a) to illustrate the effect of the inductive lumped element. As expected from Eq. (1), the absorption frequency decreases from 2.85 GHz to 2.1 GHz as *L*_c_ increases from 11 nH to 21 nH. However, when *L*_c_ is increased, the self-resonant frequency (SRF) decreases owing to the parasitic capacitance of the chip inductor. After considering the SRF, an *L*_c_ of 17 nH is determined to be required to ensure stable operation. Finally, the simulated absorptivity is achieved with a value of 99.6% at 2.4 GHz. At 2.4 GHz, the electrical size of the MM unit cell is 0.027λ × 0.027λ × 0.043λ, as shown in Fig. 4(b).

### Fabrication and Measurement Results

**Figure 5:**
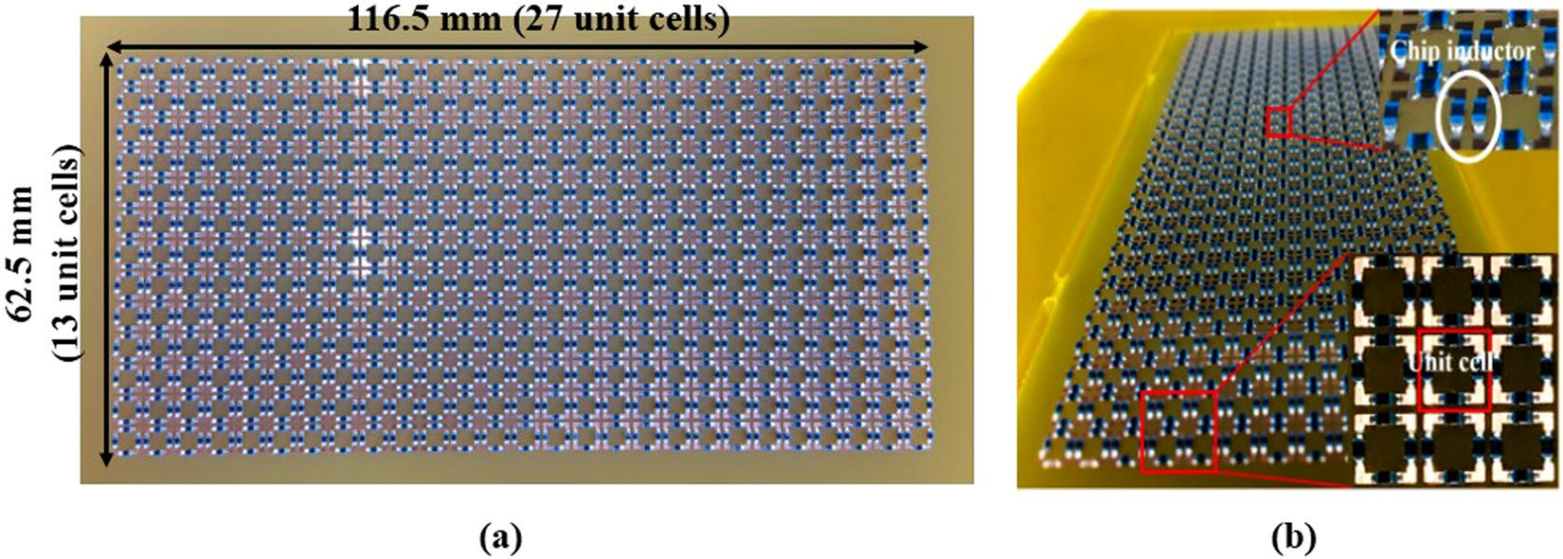
Picture of the fabricated absorber prototype: (**a**) Top layer. (**b**) Detailed structure.

A prototype with 13 × 27 unit cells is fabricated on an FR-4 substrate, as shown in Fig. 5, to experimentally demonstrate the performance of the proposed MM absorber. The overall dimensions are 116.5 mm × 62.5 mm × 5.6 mm and 1608 chip inductors are loaded using surface-mounting technology (SMT). Chip inductors of 17 nH (LQW15AN_80 Series) are used as the inductive lumped elements. An inductance tolerance of 2% is expected to generate errors of 1.2% in the absorptivity.

**Figure 6:**
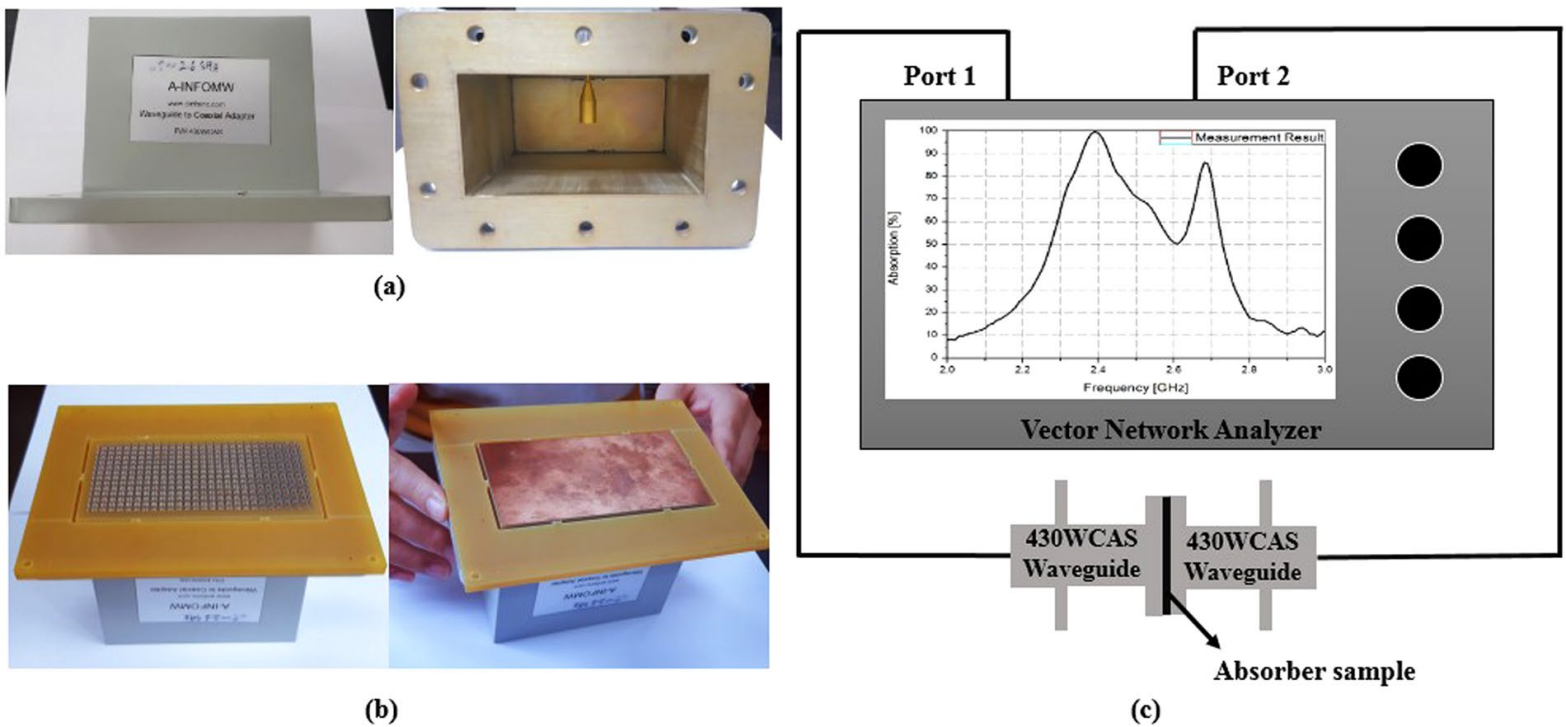
Measurement set-up for experimental verification. (**a**) CHENGDO AINFO 430WCAS waveguide for measurement. (**b**) Fabricated absorber sample on the waveguide. (**c**) Two-port measurement setup.

Figure 6 shows the measurement set-up for the experimental verification. We used an ANRITS MS2038C vector network analyser (VNA, frequency range: 5–20 GHz) and two CHENGDO AINFO 430WCAS waveguides (WR-430, Frequency range: 1.7–2.6 GHz) to measure the characteristics of the fabricated sample, as shown in Fig. 6(a). Figure 6(b) shows the fabricated absorber sample on the waveguide. We measured the absorption of the fabricated sample by loading the open aperture of the rectangular waveguides, as shown in Fig. 6(c). As the bottom side of the fabricated sample is completely covered with copper, the transmission coefficient is zero. Thus, we measured only the reflection coefficient to calculate the absorptivity.

**Figure 7:**
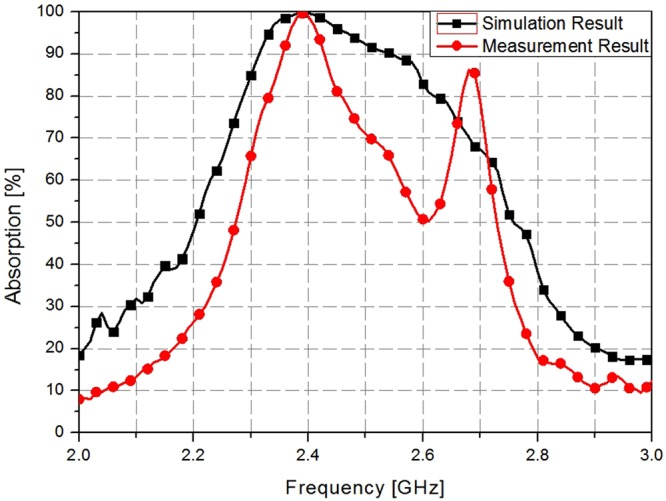
Simulated and measured absorptivity results of the proposed MM absorber.

The measured absorptivity is compared with the simulated absorptivity in Fig. 7. At 2.4 GHz, both the simulated and measured absorptivity are approximately 100%. As shown in Fig. 7, the discrepancy between the simulation and measurement occurs at 2.7 GHz because of the WR-430 frequency range limitation (frequency range: 1.7–2.6 GHz). Table 1 presents a comparison of the proposed metamaterial absorber with the previously reported low-frequency metamaterial absorbers.

**Table 1:** Comparison of the proposed MM absorber and previously reported low-frequency MM absorbers.

Reference	Number of layers	Frequency (GHz)	Thickness (mm)	Unit cell size (mm)	Electrical volume
^27^	3	10.28	0.56	2.7 × 2.7	0.0925λ × 0.0925λ × 0.0192λ
^28^	3	2	1.27	12.2 × 12.2	0.081λ × 0.081λ × 0.0086λ
^29^	2	2.5	2.4	20 × 20	0.17λ × 0.17 λ × 0.02λ
^30^	3	0.45	16	40 × 40	0.06λ × 0.06λ × 0.024λ
^31^	1	0.42	14	62.5 × 62.5	0.088λ × 0.088λ × 0.02λ
This work	1	2.4	5.6	4 × 4	0.027λ × 0.027λ × 0.043λ

## Methods

### Simulation

We used the ANSYS high-frequency structure simulator (HFSS), which is a full-wave simulator, to simulate the proposed structure. We designed the overall structure, including 13 × 27 unit cells, to have dimensions of 116.5 mm × 62.5 mm × 5.6 mm; one pair of waveguides was designed. The waveguide dimensions were referenced from the CHENGDO AINFO 430WCAS waveguide datasheet. A pair of wave ports was used in the waveguide as the excitation port. The FR-4 substrate, which has a dielectric constant of 3.9 and loss tangent of 0.02, was used for the proposed absorber. The copper conductivity was defined as 5.8 × 10^7^ S/m. Additionally, the lumped element used for the chip inductor possessed an inductance of 17 nH.

### Measurement

For experimental verification, we fabricated the prototype sample through printed circuit board (PCB) processing. The total sample dimensions were 116.5 mm × 62.5 mm × 5.6 mm. We used SMT processing to attach the chip inductors on the top layer. Chip inductors with an inductance of 17 nH (LQW15AN_80 Series) were used as the inductive lumped elements, with dimensions of 1.6 mm × 0.8 mm (1608 metric code). The bottom layer was completely covered with copper. We used the ANRITS MS2038C VNA and two CHENGDO AINFO 430WCAS waveguides to measure the prototype sample. The VNA and waveguide frequency ranges were 5 kHz to 18 GHz and 1.7–2.6 GHz, respectively. We measured the absorption of the fabricated sample by loading the open aperture of the rectangular waveguides. We measured only the reflection coefficient for calculating the absorptivity because the bottom layer is completely covered with copper.

## Discussion

We proposed a low-frequency MM absorber using a subwavelength unit cell. Owing to the proposed inductive square-loop resonator, the overall volume of the unit cell is 0.027λ × 0.027λ × 0.043λ at 2.4 GHz. Its dimensions are compared with those of previously reported low-frequency MM absorbers in Table 1. We observed that the proposed unit cell possesses much smaller dimensions than other unit cells and its realized in a single layer is an additional benefit. The performance of the proposed absorber is numerically and experimentally demonstrated. Both the simulation and measurement results indicate that perfect absorptivity is achieved at 2.4 GHz and a chip inductor tolerance of 2% yields errors of 1.2% and 1.25% in the absorptivity and absorption frequency, respectively. Because the proposed concept is easy to design and fabricate, it can be applied to ultra-low-frequency and acoustic absorber applications.
